# The Reciprocity of Church-Based Support and Psychological Well-Being: Religion, Aging and Health Survey (RAH)

**DOI:** 10.1093/geroni/igaf122.3446

**Published:** 2025-12-31

**Authors:** Landon Peeples, Lauren Chrzanowski, Benjamin Mast

**Affiliations:** University of Louisville, Louisville, Kentucky, United States; University of Louisville, Louisville, Kentucky, United States; University of Louisville, Louisville, Kentucky, United States

## Abstract

Church-based support provides both social and emotional benefits, yet little is known about its reciprocation and effects on well-being. This study uses Religion, Aging, and Health (RAH) survey data from older adults aged 65 and over within the United States to investigate (1) how receiving pastoral and congregational support influences the likelihood of providing church-based support and (2) the psychological correlates of providing supporting. Linear regression analysis (N = 813) evaluated the effects of receiving support on providing support, controlling for age, gender, race, and denomination. MANOVA (N = 1118) assessed the impact of providing support on depression, self-esteem, life satisfaction, and self-rated health. The regression model was statistically significant (R² = 0.225, F (9,803) = 25.907, p < 0.001). Receiving congregational support (B = 0.249, p = 0.002) and age (B = 0.012, p = 0.002) was positively associated with providing support. Pastoral support, gender, race, denomination, and interaction terms were not significant. MANOVA showed a significant multivariate effect (Pillai’s Trace = .018, F (4, 1113) = 4.979, p < 0.001). Providing support was associated with lower depression (F (1, 1116) = 7.410, p = 0.007), higher self-esteem (F (1, 1116) = 9.997, p = 0.002), and greater life satisfaction (F (1, 1116) = 6.857, p = 0.009) but did not impact self-rated health (F (1, 1116) = 1.037, p = 0.309). These findings highlight how providing support can influence psychological well-being within faith communities. Future research should explore how other church-based support contributes to well-being in diverse populations.

